# Efficacy of Jinshui Liujun decoction on chronic obstructive pulmonary disease patients: a systematic review and meta-analysis

**DOI:** 10.3389/fphar.2025.1567452

**Published:** 2025-05-21

**Authors:** Wanqiu Huang, Hui Wang, Di Wu, Lu Zhang, Jiabing Tong, Minghui Yu, Zegeng Li, Qinjun Yang

**Affiliations:** 1 Anhui University of Chinese Medicine, Hefei, China; 2 The First Affiliated Hospital of Anhui University of Chinese Medicine, Hefei, China; 3 Anhui Province Key Laboratory of the Application and Transformation of Traditional Chinese Medicine in the Prevention and Treatment of Major Pulmonary Diseases, Hefei, China

**Keywords:** Jinshui Liujun decoction, traditional Chinese medicine, chronic obstructive pulmonary disease, trial sequential analysis, meta-analysis

## Abstract

**Background:**

Jin Shui Liu Jun Decoction (JLD) is a classical prescription in Traditional Chinese Medicine (TCM). In recent years, JLD has shown beneficial effects on patients with chronic obstructive pulmonary disease (COPD). However, the existing clinical research results are contradictory, and high-quality, evidence-based medical evidence is lacking. Therefore, the exact therapeutic efficacy of JLD has not been fully evaluated.

**Objectives:**

This study aimed to ascertain the precise therapeutic efficacy of JLD in treating COPD.

**Data sources and Method:**

A search of 10 electronic databases was conducted up to 30 November 2024. The standardized mean difference (SMD) was used to assess continuous variables, while the relative risk (RR) was calculated to evaluate dichotomous variables. The Luis Furuya-Kanamori (LFK) asymmetry index, along with the Doi plot, Egger’s test, and Begg’s test, were used to assess publication bias. Sensitivity analysis was performed to evaluate the stability of the conclusions. Furthermore, trial sequential analysis (TSA) was used to assess the risk of false-positive results and to estimate the required sample size for the meta-analysis. Finally, single-factor and multi-factor meta-regression were conducted to analyze the sources of heterogeneity.

**Results:**

A total of 22 trials meeting the inclusion criteria were included, including 1,817 COPD participants. The meta-analysis indicated that JLD could improve overall treatment efficacy (RR = 1.15, 95% CI: 1.053–1.256, *P*_Z_ = 0.002) and pulmonary function (FEV1: SMD = 0.661, 95% CI: 0.276–1.046, *P*_*Z*_ = 0.001; FEV1% Predicted: SMD = 0.368, 95% CI: 0.067–0.669, *P*_*Z*_ = 0.017; FVC: SMD = 0.814, 95% CI: 0.392–1.235, *P*_*Z*_ < 0.001; FEV1/FVC Ratio: SMD = 0.602, 95% CI: 0.311–0.893, *P*_*Z*_ < 0.001) in COPD, potentially offering benefits in traditional Chinese medicine syndrome scores (SMD = 0.936, 95% CI: 0.301–1.571, *P*_Z_ = 0.004) and 6-min walk distance (6MWD: SMD = 0.744, 95% CI: 0.182–1.306, *P*_Z_ = 0.009). The subgroup analysis revealed a higher overall efficacy and improvement in FEV1/FVC Ratio of JLD treatment for stable chronic obstructive pulmonary disease (sCOPD) compared to the acute exacerbation of chronic obstructive pulmonary disease (AECOPD) group (SMD:0.605 v.s. 0.574, *P*_*Z*_ < 0.05). Additionally, compared to the simple JLD treatment plan, the conventional biomedicine (CBM)+JLD scheme showed superior overall efficacy in treating COPD patients (RR = 1.15, 95% CI: 1.043–1.268, *P*_Z_ = 0.005).

**Conclusion:**

Treatment with JLD can effectively improve the overall efficacy and pulmonary function (FEV1%pred requires more research for confirmation) of COPD patients. However, the methodological quality of the included trials may limit the generalizability of the study’s findings. Sources of heterogeneity were partially identified through meta-regression, but further rigorous randomized controlled trials are still required.

**Systematic Review Registration:**

https://osf.io/msw7b.

## Graphical Abstract

**Figure:**
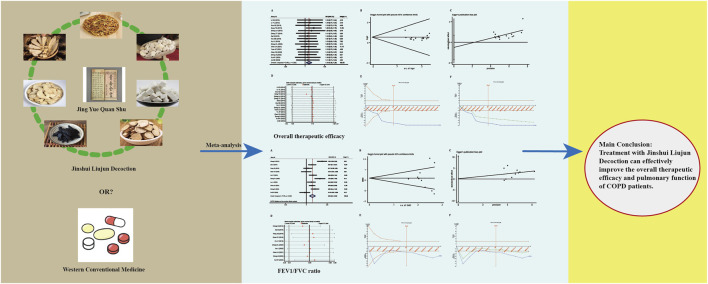

## 1 Introduction

Chronic obstructive pulmonary disease (COPD) is a globally prevalent disease characterized primarily by persistent and progressive airflow obstruction (Agustí et al., 2023). According to a survey on the burden of disease, COPD ranks among the top ten causes of mortality globally, with an age-standardized mortality rate of 45.2 per 100,000 individuals. In China, the annual death toll attributed to COPD accounts for approximately 31.1% of the global total (Ong et al., 2024; Qi et al., 2022)^,^. Considering the chronic and incurable nature of COPD, its frequent exacerbations impose a substantial societal and economic burden. In clinical practice, the primary therapeutic approach for COPD focuses on delaying the degradation of the condition. Common treatments include the use of corticosteroids, anticholinergic medications, and beta-2 agonists (Bollmeier and Hartmann, 2020). These medications are effective in managing COPD but also increase the risk of opportunistic infections and cardiovascular events (Walters et al., 2018; Michele et al., 2010; Vanfleteren et al., 2018; Linhui et al., 2021). Hence, the long-term efficacy and safety of these treatments remain controversial.

Jin Shui Liu Jun Decoction (JLD), a commonly used prescription for the treatment of COPD, was first documented in the “Jing Yue Quan Shu Xin Fang Ba Zhen”. The prescription comprises seven traditional Chinese medicinal ingredients. Randomized controlled trials revealed that treatment of COPD with modifications of JLD yielded a higher rate of therapeutic efficacy and significantly improved patients’ lung function. However, due to the limited sample size of these studies, the conclusions still lack credibility. Owing to the absence of high-quality evidence, JLD has not yet been recommended in guidelines in China (MedicineWorld Federation of Chinese Medicine Societies Specialty Committee of Internal, 2023). In addition, Traditional Chinese Medicine (TCM) treatment encompasses personalized therapy and flexible modification of prescriptions. Nevertheless, these characteristics increase clinical research variability, complicating meta-analysis and heterogeneity analysis (Fayang et al., 2023). Finally, traditional meta-analyses may be prone to Type I errors, which are false positives, and may lead to inefficient medical resources allocations due to the inability to provide the desired responsiveness index score (RIS) (Hong et al., 2016).

Therefore, this study aimed to address the aforementioned issues by conducting a meta-analysis of randomized controlled trials (RCTs) on the treatment of COPD with JLD. To provide a more comprehensive evidence base, the study employed a combination of many analytical methods, including sensitivity analysis, meta-regression, and trial sequential analysis (TSA). These approaches were integrated to generate a detailed and robust chain of evidence regarding the efficacy of JLD in treating COPD. As the first systematic evaluation of JLD’s therapeutic effects on COPD, this study not only addresses existing research gaps but also offers clinicians objective and actionable guidance for its clinical application. By objectively estimating the RIS through TSA, the study further strengthens the reliability of its conclusions, thereby contributing to evidence-based decision-making in COPD management.

## 2 Methods

Our protocol was registered in the Open Science Framework (https://osf.io/msw7b). This systematic review and meta-analysis were conducted in accordance with the “Preferred Reporting Items for Systematic Reviews and Meta-Analyses: The PRISMA Statement” (Page et al., 2021).

### 2.1 Taxonomic validation of the key botanical drug

The taxonomic validation of the key botanical drugs in JLD has been verified in the Medicinal Plant Names Services (MPNS) (https://mpns.science.kew.org/mpns-portal/) and the MYCOBANK Database (https://www.mycobank.org/), as detailed in Table 1.

**TABLE 1:** Pharmacological efficacy of Chinese botanical drugs in JLD.

Chinese botanical drugs name	Full botanical drug species name	Part of botanical drugs	Main markers or active metabolites	Efficacy
Dang Gui	Angelica sinensis (Oliv.) Diels [Apiaceae; Radix Angelicae Sinensis]	Root	Ferulic acid (Lu and Wang, 2025), Angelica sinensis polysaccharides (Ren et al., 2024)	Anti-asthmatic (Fenglong et al., 2021)^,^ anti-inflammation (Oh et al., 2019), Anti-oxidatio (Fan et al., 2020)
Di Huang	Rehmannia glutinosa (Gaertn.) Libosch. ex DC. [Orobanchaceae; Radix Rehmanniae]	Root	Rehmannia Polysaccharides (Jia et al., 2023), Acteoside (Jia et al., 2023), Catalpol (Huang et al., 2022)	Anti-oxidatio (Liu et al., 2020), regulate immunity (Luo et al., 2020)
Chen Pi	Citrus reticulata Blanco [Rutaceae; Pericarpium Citri Reticulatae]	Fruit peel	Hesperidin (Yang et al., 2025), β-Pinene (Fahmy et al., 2022), Citromitin (Ullah et al., 2024)	Anti-oxidation, anti-bacterial, anti-carcinogenesis (Job et al., 2024)
Ban Xia	Pinellia ternata (Thunb.) Makino [Araceae; Rhizoma Pinelliae]	Rhizome	24-ethylcholest-4-en-3-one (Feng et al., 2025), Pedatisectine J (Chen et al., 2025)	Anti-inflammation, anti-oxidatio (Lyu et al., 2020), promote sleep (Lin et al., 2019)
Fu Ling	Poria cocos (Schw.) Wolf [Polyporaceae; Sclerotium Poriae]	Sclerotium	Poria cocos polysaccharide (Huang et al., 2025), 3,4-seco-lanostane tetracyclic triterpenoid (Guo et al., 2025)	Anti-inflammation, anti-oxidatio (Nie et al., 2020), anti-tumor (Li et al., 2019)
Sheng Jiang	Zingiber officinale Roscoe [Zingiberaceae; Rhizoma Zingiberis Officinalis]	Rhizome	Gingerols (Rasheed, 2020), Shogaols (Kim et al., 2025), Zingerone (Wang et al., 2025)	Anti-inflammation (Ballester et al., 2022), anti-oxidatio (Kocaadam and Şanlier, 2017)
Gan Cao	Glycyrrhiza uralensis Fisch. ex DC. [Fabaceae; Radix Glycyrrhizae]	Root and rhizome	Glycyrrhizin (Li et al., 2025), Glycyrrhetinic acid (Ali et al., 2025), Isoliquiritigenin (Chen et al., 2025)	Anti-inflammation, anti-oxidatio, anti-allergy, anti-bacterial (Kwon et al., 2020)

### 2.2 Search strategy

Two researchers independently searched databases including PubMed, Embase, Web of Science, Cochrane Library, SCOPUS, Sinomed, CNKI, Proquest, Wanfang Data, and the VIP Chinese Journal Service Platform. The search period spanned from the inception of each database to 30 November 2024. To avoid omissions, the references of the included studies were manually searched when necessary. The search terms included “Jin Shui Liu Jun Jian”, “Jinshui Liujun Decoction”, and “Chronic Obstructive Pulmonary Diseases”. The detailed search strategies are provided in Supplementary Table S1.

### 2.3 Inclusion criteria

#### 2.3.1 Research type

Only randomized controlled trials evaluating the efficacy and safety of JLD in treating COPD were included, without restrictions on language, blinding, publication type, etc.

#### 2.3.2 Participants

Participants were diagnosed with COPD according to the Global Initiative for Chronic Obstructive Lung Disease or the Chinese expert consensus on the diagnosis and treatment of chronic obstructive pulmonary disease (COPD), without restrictions based on age, gender, region, or demographic characteristics.

#### 2.3.3 Type of intervention

Participants in the control group received CBM treatment. CBM encompasseds essential treatments: (1) General supportive therapy, such as (A) oxygen supplementation, (B) necessary cardiac examinations and treatments, (C) monitoring of temperature, (D) monitoring of blood pressure, (E) monitoring of blood glucose levels, (F) nutritional support; (2) Common medications for COPD treatment, such as (A) β2 receptor agonists, (B) anticholinergic drugs, (C) theophylline, (D) ICS (inhaled corticosteroids), (E) mucolytics; (3) Anti-infective therapy, primarily involving antibiotics. All treatment regimens and dosages were included in the meta-analysis.

The treatment group included patients treated with JLD alone or with modified JLD. JLD’s formulation comprises a decoction (Tang) consisting of Angelica sinensis, Rehmannia glutinosa, Citrus reticulata peel, Pinellia ternata, Poria cocos, Glycyrrhiza uralensis (baked), and fresh ginger. Modified JLD refers to JLD supplemented with additional traditional Chinese botanical drugs based on syndrome differentiation, the modified JLD, comprises similar primary ingredients and exerts similar therapeutic effects to JLD. Interventions combining JLD with CBM therapy were also included. No restrictions were imposed on the dosage and formulation of JLD; standard dose ranges were deemed acceptable, with a minimum treatment duration of 10 days.

#### 2.3.4 Outcome measures

The outcome measures included overall therapeutic efficacy, lung function, 6-min walking distance (6MWD), traditional Chinese medicine syndrome score, interleukin-6 levels, etc.

### 2.4 Exclusion criteria

This meta-analysis excluded the following studies: 1. Quasi-randomized controlled trials that allocated participants based on birth dates, medical record numbers, admission dates, or ID numbers; 2. Studies involving mechanically ventilated patients; 3. Studies involving comorbidities such as bronchial asthma, lung cancer, bronchiectasis, pulmonary hypertension, or other mental disorders/chronic diseases like depression, anxiety, or malnutrition; 4. Studies without a control group; 5. Non-randomized controlled trials (RCTs) including abstracts, reviews, case reports, expert opinions, animal experiments, etc.; 6. Studies with duplicated data; 7. Studies lacking extractable data from text/tables or obtainable data from authors; 8. Studies with significant errors in data processing and statistical methods; 9. Studies where the intervention group used external therapies or exercise therapies in addition to JLD, such as respiratory exercises, Baduanjin, Tai Chi, rehabilitation training, moxibustion, acupoint application, etc.; 10. Studies that have modified the Jin Shui Liu Jun Decoction (JLD) to include more than six botanical drugs.

### 2.5 Data extraction and quality assessment

Based on the inclusion and exclusion criteria, data extraction was independently conducted by two researchers and were subsequently cross-verified. Discrepancies were resolved through discussion or by involving a third researcher. The fundamental study details (authors, publication date, title, etc.), participant characteristics (sample size, gender, age, etc.), intervention specifics (therapy types and durations for both groups), and outcome measures (primary and secondary endpoints, adverse events, etc.) were extracted from the studies. For studies reporting multiple measurements, data from the final observation period were utilized for analysis.

The methodological quality of the retrieved research literature was evaluated using the modified Jadad scoring scale. The evaluation criteria included random sequence generation, allocation concealment, blinding, and withdrawals and dropouts. Studies were classified as low quality (1–3 points) or high quality (4–7 points), where 7 points indicating the highest quality.

### 2.6 Data analysis methods

A meta-analysis of the included studies was performed using Stata (Version 14.0) and trial sequential analysis (TSA v0.9.5.10 Beta) software. Standardized mean differences (SMD) were utilized to access continuous variables, while relative risk (RR) with 95% confidence intervals (CI) were computed for dichotomous variables. Heterogeneity among study results was evaluated using the Q test, employing a fixed-effects model when *I* ^2^ ≤ 50% and *P*_Q_ ≥ 0.10. In contrast, a random-effects model was applied when *I* ^2^ ≥ 50% and *P*_Q_ < 0.10. When the number of studies included in the analysis was less than 10, publication bias was evaluated using Doi plots and the Luis Furuya-Kanamori (LFK) asymmetry index, where LFK index values within ±1, ±1 to ±2, and ±2 or greater indicated no asymmetry, slight asymmetry, and significant asymmetry, respectively (Furuya-Kanamori et al., 2018; Li et al., 2023). When more than ten studies were included in the analysis, Egger’s test and Begg’s test were employed to assess publication bias, with *P* ≥ 0.05 indicating no significant publication bias. Sensitivity analysis was conducted using the leave-one-out method. Furthermore, subgroup analyses were performed to explore the efficacy of different treatment regimens for COPD patients and the efficacy of JLD across different stages of COPD, with each subgroup containing at least three studies. Meta-regression was employed to analyze variables that may influence the outcomes, with *P* < 0.05 indicating statistical significance. Subgroup analyses and meta-regression included at least 10 studies for each outcome measure.

## 3 Results

### 3.1 Selection and description of studies

**FIGURE 1:**
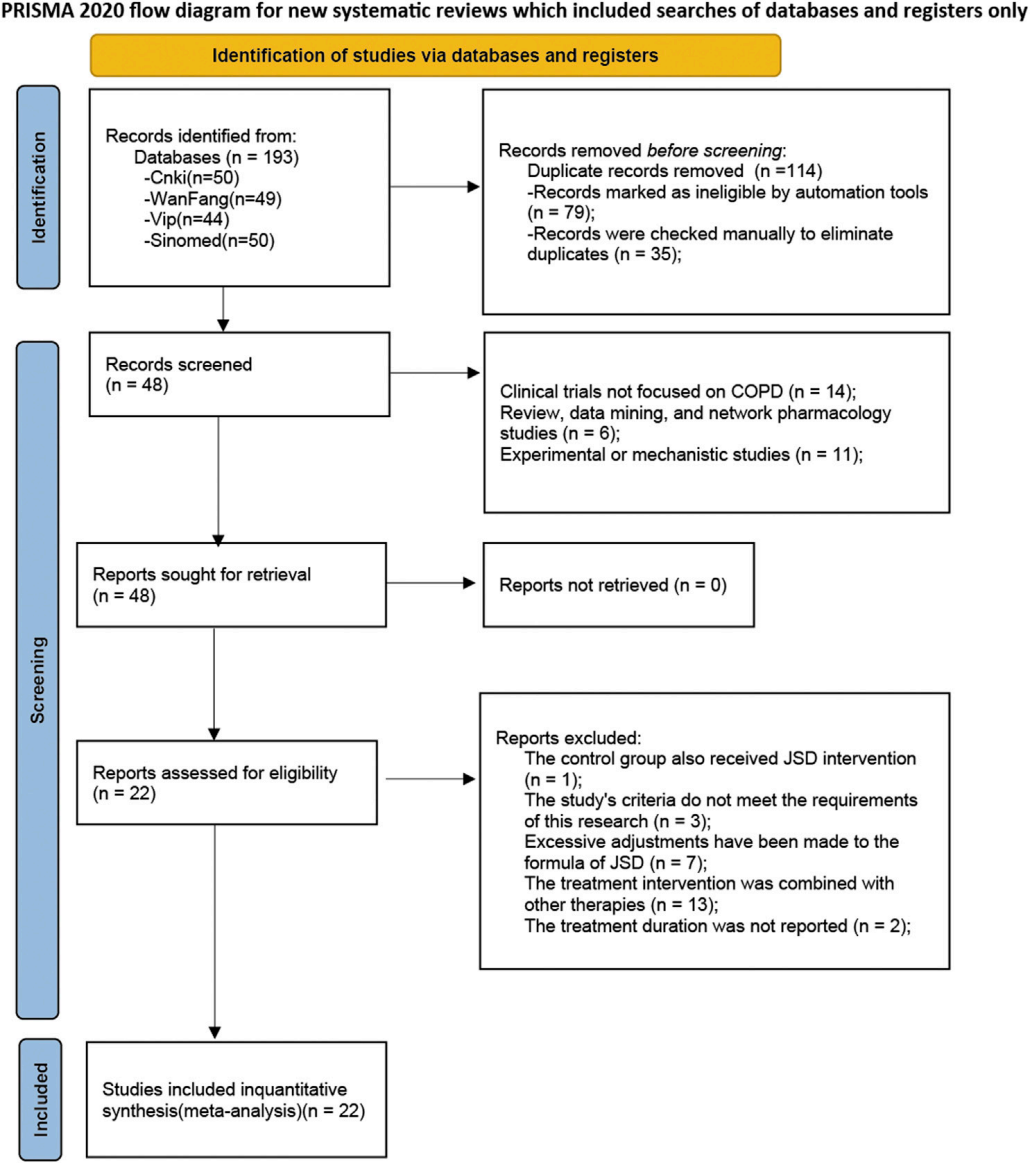
Flowchart of the screening process.

Through database searches, a total of 193 records were initially retrieved. Subsequently, 79 records identified as duplicates by the software due to multi-database searches were removed, along with 35 additional duplicates detected through manual review. The titles and abstracts were examined, and 11 records were identified as experimental or mechanistic studies, 6 records as reviews, data mining, or network pharmacology studies, and 14 records as non-COPD studies. Upon reading the full texts, one record was found to have a control group that also used JLD intervention, 3 records had study indicators that did not meet our criteria, 7 records had excessive modifications to the ingredients of JLD, 13 records combined other therapies, and 2 records did not report the treatment course. Therefore, all these records were excluded from the analysis. Finally, 22 studies were included (Xiaoqi, 2010; Rongzhao et al., 2011; Qi, 2012; Yunlong et al., 2012; Guizhen, 2013; Lihua et al., 2013; Yuqin et al., 2013; Tiantao et al., 2014; Baosong et al., 2015; Bei et al., 2015; Jingqin et al., 2015; Kaiyue et al., 2018; Liyan et al., 2019; Chuanlin, 2020; Jingqin et al., 2020; Li and Liu, 2020; Jiping, 2021; Linhui et al., 2021; Cangsong et al., 2022; Shan, 2022; Baozheng et al., 2023; Mengyao, 2023), involving 1817 participants in this meta-analysis, with 908 cases in the experimental group and 909 cases in the control group. The inclusion and exclusion process is displayed in Figure 1.

All the studies were published between 2010 and 2023, with treatment durations ranging from 10 days to 6 months. Fourteen studies focused on the stable phase of COPD, 2 studies on AECOPD, and 6 studies did not specify and the disease condition could not be determined based on their content. Only one study did not mention the dosage form, while the remaining studies all used decoction for administration. All the experimental groups received JLD or JLD combined with CBM treatment, while the control groups received CBM treatment. Sixteen studies reported the overall efficacy rate; seven studies reported forced expiratory volume in one second (FEV1); eleven studies reported the ratio of FEV1 to forced vital capacity (FVC), which is expressed as FEV1/FVC(%); five studies reported FEV1%predicted; and four studies each reported FVC, TCM syndrome scores, 6MWT, and IL-6. Three studies reported dropouts and losses to follow-up, 3 studies reported whether adverse events occurred, and 1 study reported follow-up conditions. All details of the included studies can be found in Table 2.

**TABLE 2:** Fundamental characteristics of included literature.

Study author, year	Outcome Measures^a^	COPD stage	Intervention measures		Sample size		Therapy duration	JADA	Gender (M,F)		Age		Duration	
			T	C	T	C			T	C	T	C	T	C
Li XQ (2010)	①	Not Mentioned	JLD	CBM	32	31	20D	2	(18/14)	(17/14)	62.5	61	15	14
Zhang RZ (2011)	③⑤⑦	sCOPD	CBM + JLD	CBM	43	45	24W	3	(36/7)	(39/6)	56.20 ± 7.12	55.21 ± 7.01	16.02 ± 8.96	15.26 ± 9.10
Chang Q (2012)	⑤	sCOPD	JLD	CBM	42	42	20D	2	(26/16)	(24/18)	65.6 ± 6.3	64.7 ± 6.1	14.4 ± 2.6	15.1 ± 2.7
Lv YL (2012)	①	Not Mentioned	JLD	CBM	32	31	20D	2	(18/14)	(15/17)	62.5	61	15	14
Guo GZ (2013)	①	Undetermined	JLD	CBM	31	27	20D	2	(16/15)	(14/13)	62.29 ± 13.66	67.60 ± 11.99	NA	NA
Song YQ (2013)	①	sCOPD	CBM + JLD	CBM	100	100	30D	2	(64/36)	(58/42)	70.5 ± 7.3	69.5 ± 6.8	28.5 ± 5.3	30 ± 2.3
Zhang LH (2013)	①	Not Mentioned	CBM + JLD	CBM	29	29	30D	3	(18/11)	(20/9)	68.5 ± 7.3	64.5 ± 6.8	28.5 ± 5.3	30.0 ± 2.3
Zhang TT (2014)	①	sCOPD	CBM + JLD	CBM	30	30	30D	2	M31	F29	49 to 80		10 to 25	
Dai B (2015)	①③⑤⑦	Not Mentioned	CBM + JLD	CBM	50	50	14D	2	(26/24)	(27/23)	62.5	61.8	15.6	14.7
Hou BS(2015)	①	sCOPD	CBM + JLD	CBM	39	40	15D	2	M45	F34	40 to 70		5 to 30	
Peng JQ (2015)	②⑤	sCOPD	CBM + JLD	CBM	38	38	180D	4	(29/9)	(28/10)	64.53 ± 4.95	63.79 ± 6.08	18.95 ± 7.77	21.55 ± 7.92
Guan KY(2018)	②③⑥⑤	sCOPD	CBM + JLD	CBM	30	30	6M	2	(25/5)	(25/5)	68.80 ± 6.18	71.83 ± 7.34	7.76 ± 3.87	10.29 ± 6.08
Xu LY (2019)	①②④⑤⑥⑦	sCOPD	CBM + JLD	CBM	53	53	4W	2	(33/20)	(32/21)	63.86 ± 5.03	64.13 ± 5.62	NA	NA
Peng JQ (2020)	⑥ ⑧	Undetermined	CBM + JLD	CBM	22	22	6M	2	(12/10)	(13/9)	53.12 ± 15.33	53.06 ± 15.58	6.05 ± 2.41	6.11 ± 2.34
Zhang CL (2020)	①⑤⑦	sCOPD	CBM + JLD	CBM	34	34	14D	3	(26/8)	(27/7)	72 [62, 75]	72 [68,78]	NA	NA
Luo L (2020)	②⑤	sCOPD	CBM + JLD	CBM	45	44	6M	3	(26/19)	(27/17)	58.32 ± 5.13	58.42 ± 5.49	16.54 ± 6.02	16.98 ± 6.46
Chen LH (2021)	①②③⑤	sCOPD	CBM + JLD	CBM	80	80	20D	2	(50/30)	(46/34)	69.47 ± 7.23	68.63 ± 8.21	9.23 ± 2.41	8.86 ± 3.68
Yuan JP (2021)	①	sCOPD	CBM + JLD	CBM	30	30	3M	3	Total (41/19)		Total (65.29 ± 4.71)		NA	NA
Chen CS(2022)	①②③④⑧	AECOPD	JLD	CBM	40	45	8W	3	(30/10)	(32/13)	65.86 ± 7.28	64.31 ± 7.06	10.05 ± 2.09	10.23 ± 2.16
Zhong S (2022)	①②④⑤⑧	sCOPD	CBM + JLD	CBM	50	50	2M	3	(30/20)	(27/23)	60.28 ± 4.14	60.06 ± 4.29	9.15 ± 1.57	9.97 ± 1.63
Xu MY(2023)	①⑤⑥	sCOPD	CBM + JLD	CBM	28	28	3M	4	(17/11)	(18/10)	64.30 ± 8.56	63.07 ± 9.19	NA	NA
Jin BZ (2023)	①②④⑧	AECOPD	CBM + JLD	CBM	30	30	10D	2	(21/9)	(23/7)	53 to 81	56 to 78	3 to 20	3 to 19

^a^ Note: ①: Overall Therapeutic Efficacy; ②: FEV1; ③: FEV1% Predicted; ④:FVC; ⑤: FEV1/FVC, ratio; ⑥: TCM, syndrome scores; ⑦:6MWD; ⑧IL-6.

### 3.2 Effects of JLD on overall therapeutic efficacy

**FIGURE 2:**
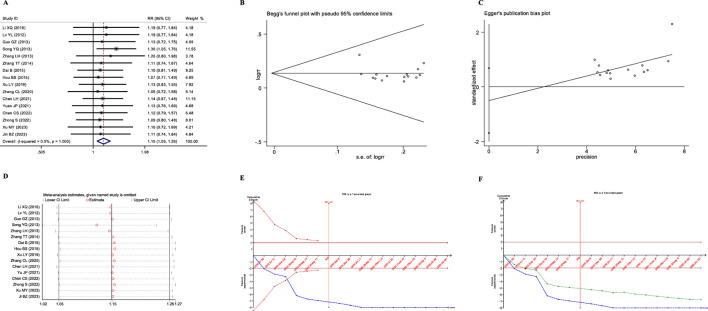
Meta-analysis diagrams of overall therapeutic efficacy. **(A)** Forest plot. **(B)** Begg's funnel plot. **(C)** Egger's publication bias plot. **(D)** Sensitivity analysis. **(E)** TSA analysis. **(F)** Penalized statistical analysis.

A total of 16 studies reported the overall therapeutic efficacy of JLD in the treatment of COPD, including a total of 1,376 patients (688 in both the experimental group and the control group, Figure 2A).No significant heterogeneity was observed among the studies (*I* ^2^ = 0.000, *P*_Q_ = 1), and a fixed-effects model was used for the meta-analysis, which showed a significantly higher overall therapeutic efficacy in the experimental group compared to the control group (RR = 1.15, 95% CI: 1.053–1.256, *P*_Z_ = 0.002). The Egger test and Begg test indicated that the sample distribution was essentially symmetrical, with P values greater than 0.05 (*P*_Egger_ = 0.381, *P*_Begg_ = 0.28, Figures 2B,C), suggesting no publication bias. Sensitivity analysis revealed that the conclusion was stable (Figure 2D). Subgroup analysis indicated that the therapeutic effect of JLD in treating stable COPD was more significant compared to AECOPD (RR = 1.153, *P* = 0.011, Table 3). Moreover, compared with the JLD group, the CBM + JLD group achieved superior therapeutic effects (RR = 1.15, *P* = 0.005, Table 3).

**TABLE 3:** Subgroup Analysis of overall therapeutic efficacy.

Subgroup	Number of studies	RR	95%CI	Heterogeneity		Z	*P*
				*I* ^*2*^	*P*_*Q*_		
sCOPD	9	1.153	(1.033,1.287)	0%	0.978	2.54	0.011
AECOPD	2	1.112	(0.858,1.441)	0%	0.972	0.8	0.423
JLD	4	1.151	(0.939,1.411)	0%	0.994	1.36	0.175
JLD + CBM	12	1.15	(1.043,1.268)	0%	0.997	2.8	0.005

The TSA results showed that the cumulative Z-curve crossed the conventional boundary after the inclusion of the first study, crossed the TSA boundary after the inclusion of the fourth study, and the sample size exceeded the RIS (RIS: 455) after the inclusion of the seventh study (Figure 2E). Further penalized statistical analysis indicated that the penalized Z-curve crossed the conventional boundary after the inclusion of the second study (Figure 2F). Both the results of the meta-analysis and TSA confirmed that JLD effectively improved the overall therapeutic efficacy of the treatment for COPD.

### 3.3 Meta-analysis of pulmonary function

#### 3.3.1 Effects of JLD on FEV1 of COPD patients

**FIGURE 3:**
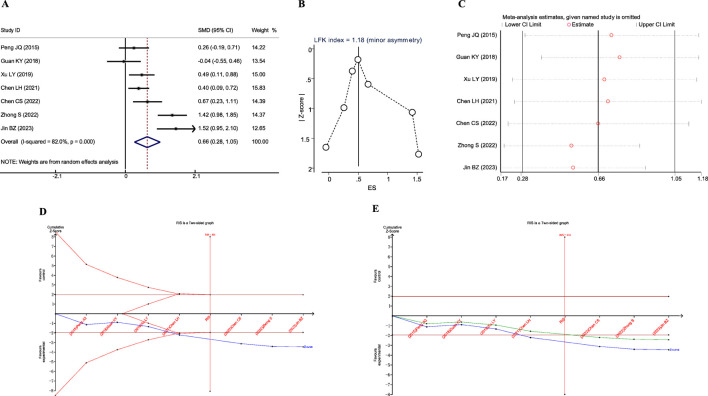
Meta-analysis diagrams of FEV1. **(A)** Forest plot. **(B)** Doi plot. **(C)** Sensitivity analysis. **(D)** TSA analysis. **(E)** Penalized statistical analysis.

A total of seven studies reported the impact of JLD treatment on the FEV1 of patients with COPD. One study was excluded due to significant data errors (Li and Liu, 2020). The remaining seven studies involved 647 patients (321 in the experimental group and 326 in the control group). Heterogeneity was found among the studies (*I* ^2^ = 82%, *P*_Q_ < 0.001), and a random-effects model was employed for the meta-analysis. The results showed that the therapeutic effect of the experimental group on improving pulmonary function, specifically FEV1, was significantly superior to that of the control group (SMD = 0.661, 95% CI: 0.276–1.046, *P*_Z_ = 0.001), as shown in Figure 3A.

The Doi plots displayed slight asymmetry (LFK index = 1.18, Figure 3B), suggesting the possibility of publication bias. Sensitivity analysis confirmed the stability of the conclusions (Figure 3C). The TSA results revealed that the cumulative Z-curve crossed both the conventional boundary and the TSA-adjusted boundary after the inclusion of the fourth study, and the sample size had reached the required information size (RIS: 416, Figure 3D). Further penalized statistical analysis indicated that the penalized Z-curve surpassed the conventional boundary after the inclusion of the fifth study (Figure 3E). Both the meta-analysis and TSA results confirmed that JLD treatment in COPD patients can improve FEV1.

#### 3.3.2 Effects of JLD on FEV1% predicted of COPD patients

**FIGURE 4:**
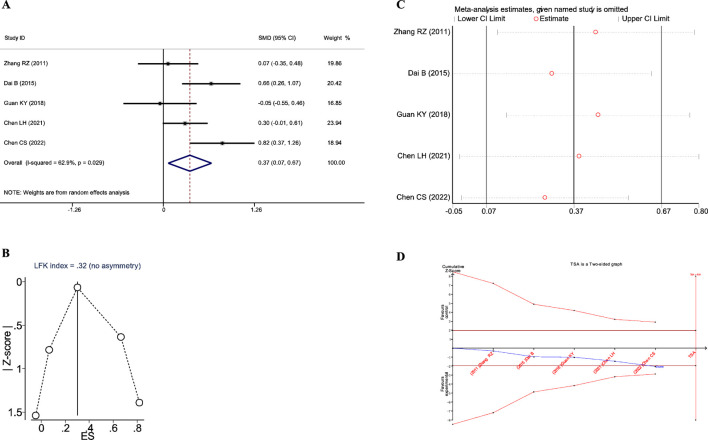
Meta-analysis diagrams of FEV1% Predicted. **(A)** Forest plot. **(B)** Doi plot. **(C)** Sensitivity analysis. **(D)** TSA analysis.

A total of five studies reported the impact of JLD treatment on the FEV1% predicted in COPD patients, including 493 patients (243 in the experimental group and 250 in the control group). Heterogeneity was observed among the studies (*I* ^2^ = 62.9%, *P*_Q_ = 0.029), and a random-effects model was utilized for the meta-analysis. The meta-analysis results indicated a significantly higher efficacy in improving pulmonary function, specifically FEV1% predicted, in the experimental group to the control group (SMD = 0.368, 95% CI: 0.067–0.669, *P*_Z_ = 0.017), as depicted in Figure 4A.

The Doi plots demonstrated no asymmetry (LFK index = 0.32, Figure 2B), suggesting no publication bias. Furthermore, sensitivity analysis confirmed the stability of the conclusions (Figure 2C). The TSA revealed that the cumulative Z-curve crossed the conventional boundary after the first study. However, the cumulative Z-curve did not cross the TSA-adjusted boundary, and the required information size (RIS: 930) was not achieved. These findings suggest that further research is needed to confirm the efficacy of JLD treatment on the FEV1% Predicted of COPD patients.

#### 3.3.3 Effects of JLD on FVC of COPD patients

**FIGURE 5:**
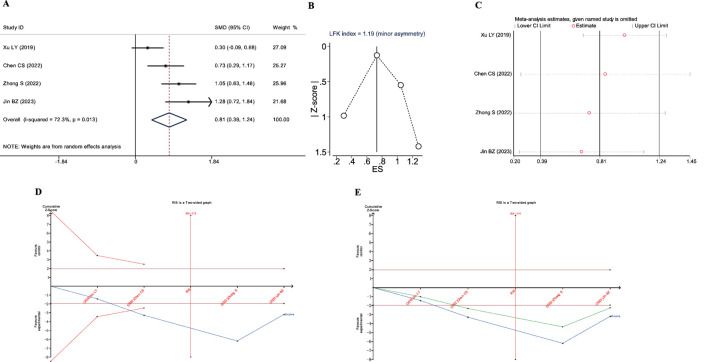
Meta-analysis diagrams of FVC. **(A)** Forest plot. **(B)** Doi plot. **(C)** Sensitivity analysis. **(D)** TSA analysis. **(E)** Penalized statistical analysis.

A total of four studies reported the impact of JLD treatment on the FVC in COPD patients, including 351 patients (173 in the experimental group and 178 in the control group). Heterogeneity was observed among the studies (*I* ^2^ = 72.3%, *P*_Q_ = 0.013), and a random-effects model was applied for the meta-analysis. The meta-analysis results revealed that the therapeutic effect of the experimental group on improving lung function, specifically FVC, was significantly superior to that of the control group (SMD = 0.814, 95% CI: 0.392–1.235, *P*_Z_<0.001), as shown in Figure 5A.

The Doi plots displayed slight asymmetry (LFK index = 1.19, Figure 5B), suggesting the possibility of publication bias. Sensitivity analysis confirmed the stability of the conclusions (Figure 5C). The TSA results showed that the cumulative Z-curve crossed both the conventional boundary and the TSA-adjusted boundary after the inclusion of the second study, and the sample size had reached the required information size (RIS: 278, Figure 5D). In addition, the penalized statistical analysis indicated that the penalized curve surpassed the conventional boundary after the inclusion of the second study, further confirming the conclusion (Figure 5E). Both the meta-analysis and TSA results substantiated that JLD treatment for COPD can improve FVC.

#### 3.3.4 Effects of JLD on FEV1/FVC ratio of COPD patients

**FIGURE 6:**
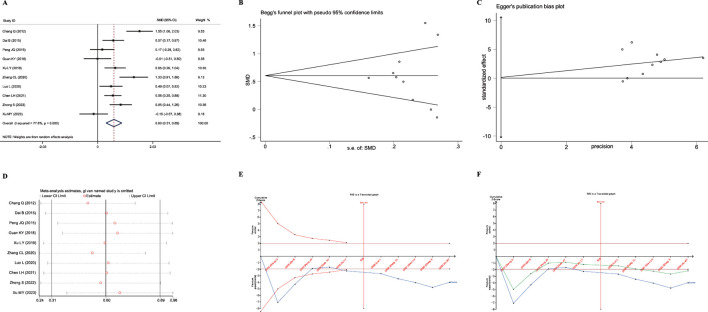
Meta-analysis diagrams of FEV1/FVC Ratio. **(A)** Forest plot. **(B)** Begg's funnel plot. **(C)** Egger's publication bias plot. **(D)** Sensitivity analysis. **(E)** TSA analysis. **(F)** Penalized statistical analysis.

A total of 10 studies reported the impact of JLD treatment on the FEV1/FVC ratio in COPD patients, involving 899 patients (450 in the experimental group and 449 in the control group). Heterogeneity was present among the studies (*I* ^2^ = 77.8%, *P*_Q_ < 0.001), so a random-effects model was employed for the meta-analysis. The meta-analysis results indicated a significantly higher improvement in the lung function ratio, specifically FEV1/FVC, in the experimental group compared to the control group (SMD = 0.602, 95% CI: 0.311–0.893, *P*_Z_ < 0.001), as shown in Figure 6A. Further subgroup analysis confirmed the therapeutic effect of JLD on the FEV1% of patients with AECOPD (SMD = 0.574, *P* = 0.005) and sCOPD (SMD = 0.605, *P* < 0.001) (Table 4). Compared with the control group, both the CBM + JLD group (SMD = 0.521, *P* = 0.001) and the JLD group (SMD = 1.546, *P* < 0.001) significantly improved the patients’ FEV1/FVC ratio (Table 4).

**TABLE 4:** Subgroup Analysis of the effects of JLD on FEV1/FVC Ratio of COPD patients.

Subgroup	Number of studies	SMD	95%CI	Heterogeneity		Z	*P*
				*I* ^*2*^	*P*_*Q*_		
sCOPD	9	0.605	(0.276,0.933)	80.3%	0.000	3.61	0.000
AECOPD	1	0.574	(0.174,0.974)	—	—	2.81	0.005
JLD	1	1.546	(1.057,2.035)	—	—	6.2	0.000
JLD + CBM	9	0.505	(0.252,0.758)	68%	0.002	3.26	0.000

The Egger test and Begg test showed that the sample distribution was essentially symmetrical, with P values greater than 0.05 (*P*_Egger_ = 0.975, *P*_Begg_ = 0.592); these results suggested no publication bias, as shown in Figures 5B,C. Sensitivity analysis confirmed the stability of the conclusions (Figure 5D). Moreover, TSA results showed that the cumulative Z-curve crossed both the conventional boundary and the TSA-adjusted boundary after the inclusion of the fifth study, with the sample size reaching the required information size (RIS: 445). Further penalized statistical analysis indicated that the penalized curve surpassed the conventional boundary after the inclusion of the eighth study, confirming the conclusion. Both the meta-analysis and TSA results provided evidence that JLD treatment for COPD can improve the FEV1/FVC ratio in patients.

#### 3.3.5 Effects of JLD on TCM syndrome scores of COPD patients

**FIGURE 7:**
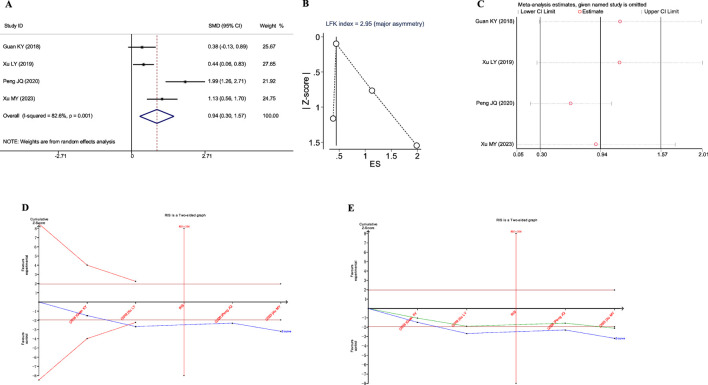
Meta-analysis diagrams of TCM Syndrome Scores. **(A)** Forest plot. **(B)** Doi plot. **(C)** Sensitivity analysis. **(D)** TSA analysis. **(E)** Penalized statistical analysis.

A total of four studies reported the impact of JLD on the total TCM syndrome scores in COPD patients, involving 266 patients (133 in both the experimental and control groups). Heterogeneity was observed among the studies (*I* ^2^ = 82.6%, *P*_Q_ = 0.001), and a random-effects model was adopted for the meta-analysis. The meta-analysis results showed that the experimental group yielded a significantly higher improvement in TCM syndrome scores compared to the control group (SMD = 0.936, 95% CI: 0.301–1.571, *P*_Z_ = 0.004), as depicted in Figure 7A.

The Doi plots exhibited significant asymmetry (LFK index = 2.95, Figure 7B), suggesting the potential presence of publication bias. Sensitivity analysis confirmed the stability of the conclusions (Figure 7C). The TSA results indicated that the cumulative Z-curve crossed the conventional boundary after the inclusion of the second study and had already reached the required information size (RIS: 206, Figure 7D). Further penalized statistical analysis indicated that the penalized curve surpassed the conventional boundary after the inclusion of the fourth study, further confirming the conclusion (Figure 7E). Both the meta-analysis and TSA results substantiated that JLD treatment for COPD can improve TCM syndrome scores in patients.

#### 3.3.6 Effects of JLD on 6MWD of COPD patients

**FIGURE 8:**
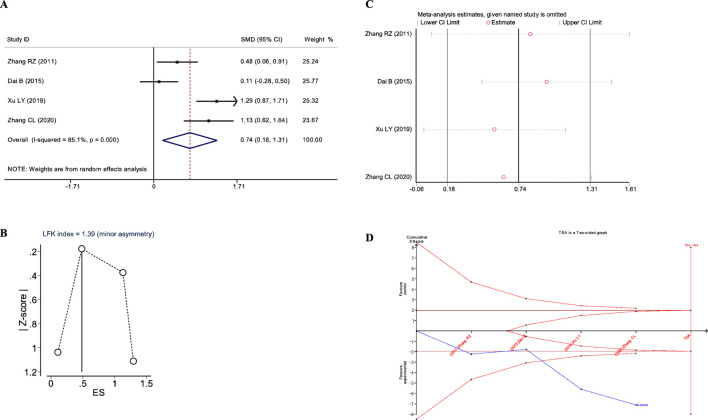
Meta-analysis diagrams of 6MWD. **(A)** Forest plot. **(B)** Doi plot. **(C)** Sensitivity analysis. **(D)** TSA analysis.

A total of four studies reported the impact of JLD on the 6MWD in COPD patients, including 362 subjects (treatment group 180, control group 182). Significant heterogeneity was observed (*I* ^2^ = 85.1%, *P*_Q_<0.001), prompting the use of a random-effects model for meta-analysis. The results indicated a statistically significant improvement in 6MWD in the treatment group (SMD = 0.744, 95% CI: 0.182–1.306, *P*_Z_ = 0.009), as illustrated in Figure 8A. This suggests that JLD can effectively enhance the 6MWD in COPD patients.

The Doi plots asymmetry test indicated slight asymmetry (LFK index = 1.39, Figure 8B), suggesting potential publication bias. Furthermore, sensitivity analysis confirmed the stability of the conclusions (Figure 8C). TSA indicated that the cumulative Z-curve crossed the conventional boundary after the first study and the TSA-adjusted boundary after the third study; however, the RIS was not reached (RIS: 404, Figure 8D). These findings suggest that further research is needed to confirm the efficacy of JLD treatment on the FVC of COPD patients.

#### 3.3.7 Effects of JLD on IL-6 of COPD patients

**FIGURE 9:**
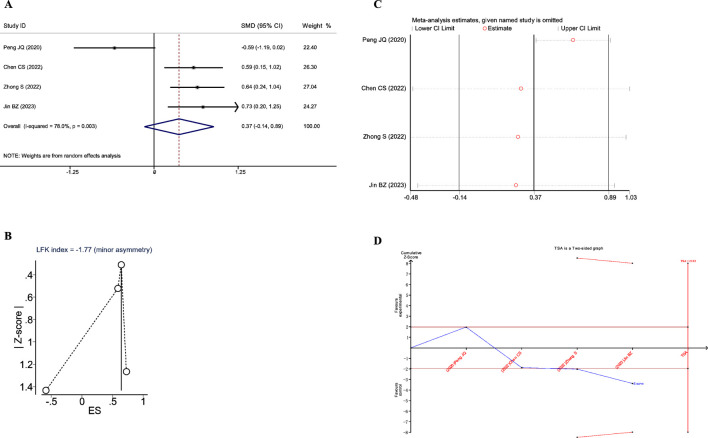
Meta-analysis diagrams of IL-6. **(A)** Forest plot. **(B)** Doi plot. **(C)** Sensitivity analysis. **(D)** TSA analysis.

Four studies reported the impact of JLD treatment on IL-6 levels in COPD patients, including 289 subjects (treatment group 142, control group 147). Significant heterogeneity was observed among the studies (*I* ^2^ = 78%, *P*_Q_ = 0.003), leading to the use of a random-effects model was adopted for the meta-analysis. The meta-analysis results indicated no statistically significant difference in improving IL-6 between the treatment and control groups (SMD = 0.372, 95% CI: −0.143 to 0.887, *P*_Z_ = 0.156), as shown in Figure 9A.

The Doi plots showed significant asymmetry (LFK index = −1.77, Figure 9B), suggesting the possibility of publication bias. Sensitivity analysis demonstrated that the conclusions were stable (Figure 9C). Moreover, the TSA results indicated that the cumulative Z-value crossed the conventional boundary after the third study but did not cross the TSA-adjusted boundary and did not reach the RIS (RIS: 2102, Figure 9D). These findings suggest that further research is needed to confirm the impact of JLD on IL-6 in COPD patients.

### 3.4 Meta-regression

#### 3.4.1 Univariate meta-regression

The modification of botanical drugs in TCM prescriptions (in the following table, “modification” is used as a substitute) and the duration of treatment in each study were taken as independent variables, and the FEV1/FVC (expressed as a percentage) as the dependent variable. Univariate meta-regression was employed to further explore the sources of heterogeneity. The results of the univariate meta-regression indicated that the “modification” and “treatment duration” were not significantly influenced the effect of JLD on FEV1/FVC (*P* < 0.05) (Table 5).

**TABLE 5:** Univariate meta-regression analysis of the FEV1/FVC.

Independent variable	Control group	Exp(b)	SE	(95%CI)	P
Modification*	2				
1		0.379	0.188	(0.117,1.228)	0.092
3		0.303	0.161	(0.086,1.065)	0.06
Treatment duration^#^	3				
1		2.437	1.072	(0.861,6.899)	0.083
2		1.215	0.572	(0.399,3.67)	0.692

*For the Modification group, “1” indicates the original prescription, “2” indicates a variation of “1–3” ingredients based on the original prescription, and “3” indicates a variation of “4–6” ingredients based on the original prescription; #For the Treatment course group, “1” indicates within one to 2 months, “2” indicates two to 3 months, and “3” indicates more than 3 months.

### 3.5 Multivariate meta-regression

A multivariate meta-regression was conducted with treatment duration, patient condition severity at inclusion (in the following table, “condition” is used as a substitute), the modification of botanical drugs in TCM prescriptions (in the following table, “modification” is used as a substitute) and treatment protocols in the treatment group as independent variables; the FEV1/FVC (expressed as a percentage) was set as the dependent variable. Considering the collinearity between the independent variables “modification” and “treatment plan” in the treatment group, two separate analyses were performed. The results indicated that treatment duration significantly influenced the FEV1/FVC (*P* < 0.05) (Tables 6, 7).

**TABLE 6:** Multivariate meta-regression analysis of the FEV1/FVC ①.

Independent variable	Exp(b)	SE	(95%CI)	*P*
condition	0.731	0.313	(0.256,2.087)	0.492
modification	1.171	0.311	(0.611,2.243)	0.574
treatment duration	2.285	0.608	(1.191,4.383)	0.021

**TABLE 7:** Multivariate meta-regression analysis of the FEV1/FVC ②.

Independent variable	Exp(b)	SE	(95%CI)	*P*
Condition	0.802	0.258	(0.365,1.764)	0.519
Treatment plan	0.472	0.167	(0.198,1.122)	0.078
Treatment duration	1.904	0.408	(1.127,3.218)	0.024

## 4 Discussion

To date, multiple studies have demonstrated the therapeutic effects of JLD on COPD; however, high-quality evidence-based medical evidence remains scarce. The only meta-analysis conducted to date included only eight studies, and was limited by a small sample size and the absence of subgroup analysis, which have constrained the generalizability of the conclusions. This study represents the first large-scale meta-analysis of JLD treatment for COPD, aiming to evaluate the therapeutic efficacy of JLD and provide a reliable reference for clinical application.

In terms of overall efficacy, this study yielded surprising results. JLD was found to enhance the overall efficacy of COPD treatment, with no evidence of publication bias. The possibility of a false positive was ruled out by TSA, reaching RIS, thereby further confirming the therapeutic effect of JLD. Subgroup analyses revealed that JLD is particularly effective in treating sCOPD and shows better efficacy when combined with CBM. The conclusions regarding AECOPD and sole JLD treatment were not statistically significant, which may be attributed to a lack of relevant studies.

Irreversible airflow limitation is a key characteristic of COPD (Yanqin et al., 2024), and pulmonary function plays a crucial role in assessing the severity of COPD (Yahong, 2022) and guiding personalized medication for COPD patients (Global Initiative for Chronic Obstructive Lung Disease, 2024). This study conducted a comprehensive analysis of the relevant published literature to evaluate the impact of JLD on pulmonary function indicators in COPD patients, including FEV1, FEV1%pred, FVC, and FEV1/FVC(%). The results showed that JLD significantly improved these indicators. However, subgroup analyses indicated that JLD had a positive effect on FEV1% in both AECOPD and sCOPD patients, and the CBM + JLD combined therapy as well as JLD monotherapy could enhance FEV1%. Still, given the limited number of studies, further research is required to confirm the efficacy in AECOPD patients and the JLD monotherapy approach.

TCM syndrome scores reflect COPD patients’ conditions, including cough, phlegm, dyspnea, and quality of life. The meta-analysis results suggest that JLD significantly improves TCM syndrome scores, and TSA indicates that RIS was achieved. However, significant publication bias was observed, and the Deeks’ funnel plot showed significant asymmetry. Considering that TCM syndrome scores are based on questionnaires, the results are influenced by patients’ and researchers’ subjectivity, which may contribute to the publication bias.

Previous studies reported that COPD can lead to osteoporosis (Fiorentino et al., 2020), muscle dysfunction (Peñailillo et al., 2022) and even atrophy in patients, potentially impairing exercise function. The 6MWD reflects the exercise capacity of COPD patients and is a strong prognostic factor (Perez et al., 2019). Despite the encouraging preliminary results, TSA analysis indicated that additional studies are required to confirm the stability and reproducibility of these findings. Furthermore, the potential presence of publication bias in existing studies may affect a comprehensive assessment of JLD efficacy.

Inflammatory responses are integral to the progression of COPD. Chronic inflammation leads to airway remodeling and exacerbates hypoxia (Barnes, 2016). Prolonged hypoxic conditions, in turn, promote inflammation, further contributing to airway remodeling (Berggren-Nylund et al., 2023), thereby creating a vicious cycle of “inflammation-airway remodeling-hypoxia-inflammation”. Aslani MR et al. found significantly higher serum IL-6 levels in the COPD population compared to healthy individuals (Aslani et al., 2022). Nicolai Obling et al. reported that inflammatory cytokines such as IL-6 were closely related to the progression of COPD (Obling et al., 2022). In addition, the present study revealed that JLD can improve IL-6 levels in COPD patients. However, TSA analysis suggests that more research is needed for confirmation, and the current evidence may be subject to publication bias. Notably, inflammatory cytokines may be the focus of clinical observation in future JLD treatment for COPD.

With the evolution of the medical treatment system, individualized disease management has become increasingly important (Brandsma et al., 2020). The greatest advantage of TCM is its flexible and adaptable prescription formulation, which allows for individualized treatment (Aijun et al., 2024). Clinical use of JLD encompasses various scenarios. First, slight variations in the TCM prescriptions were observed due to different TCM syndrome types. Second, enrolled patients may be in different disease stages, such as the acute exacerbation phase and the stable phase. Third, the duration of JLD treatment varied due as no standardized protocol has been established. Lastly, interventions in the two groups differed across studies. For instance, Chen Cangsong et al. used JLD alone for treatment, while Jin Baozheng et al. adopted an integrated TCM and biomedicine approach. Based on these discrepancies, some indicators in this study exhibited high heterogeneity. Considering that multiple subgroup analyses may increase the probability of Type I errors and the use of Bonferroni correction may lead to insufficient statistical power (Kai, 2023), univariate and multivariate meta-regressions were combined. The “treatment duration,” “the modification of botanical drugs in TCM prescriptions,” “disease condition,” and “treatment plan” were incorporated into the regression equation. The analysis revealed that “treatment duration” was the main cause of heterogeneity (*P* > 0.05). Additionally, considering the potential collinearity between “the modification of botanical drugs in TCM prescriptions” and “treatment plan”, two multivariate meta-regressions were conducted, and revealing that the conclusions remained unchanged. This suggests that future research design should focus on “treatment duration” to facilitate the production of high-quality clinical evidence.

Although the Egger test is considered more sensitive to small samples compared to the Begg test (Zhiqiang, 2013; Jie et al., 2022), some studies have indicated a low sensitivity is lower, especially with fewer than five studies (only 18.5%) (Furuya-Kanamori et al., 2018). To avoid incorrect estimation of publication bias that could affect the interpretation of the results, the LKF index and the doi plots were used to assess publication bias for outcomes with fewer than ten included studies (Li et al., 2023). The doi plots illustrated the relationship between the effect size and the normality quantile (Z-score), which helps in more intuitively identifying and assessing the asymmetry of study effects. Combined with the LKF index, which quantifies asymmetry by comparing the area differences between the two sides of the Deeks’ funnel plot, publication bias can be evaluated more accurately in small-sample outcomes. In this study, FEV1, FEV1%pred, FVC, TCM syndrome scores, 6MWD, and IL-6 were assessed using this method, and publication bias was found in all indicators except for FEV1%pred.

In terms of safety, three of the included studies specifically reported whether adverse events occurred, including cardiovascular incidents and withdrawals due to intolerance to the strong or distinctive odor of the botanical drug decoction (Rongzhao et al., 2011; Yunlong et al., 2012; Mengyao, 2023). Both were unrelated to JLD. No significant reports of adverse reactions or drug interactions related to JLD were identified in the remaining studies. Based on current clinical experience, the botanical drugs in JLD are mild in nature and demonstrate a favorable safety profile (Baozheng et al., 2023; Mengyao, 2023).

While the majority of conclusions of this study support the positive effects of JLD on the treatment of COPD, several limitations are noted: ① All included literature originated from the Chinese region, which may bias the conclusions towards beneficial effects; ② Most of the literature included in this study only mentioned “randomization” but provided no detail concerning the method of random sequence generation, potentially contributing to high heterogeneity in conclusions; ③ Few included studies reported patients’ GOLD staging. Although this study conducted meta-regression with patient conditions as a factor, the conclusions remained unchanged. However, we still believe that the therapeutic effects of JLD may vary among patients with different GOLD stages.

In summary, this study demonstrates that treatment with JLD can effectively improve the overall response rate and pulmonary function in COPD patients. Further clinical research is needed to confirm these findings.

## Data Availability

The raw data supporting the conclusions of this article will be made available by the authors, without undue reservation.
