# Correction: Efficacy of Jinshui Liujun decoction on chronic obstructive pulmonary disease patients: a systematic review and meta-analysis

**DOI:** 10.3389/fphar.2025.1643998

**Published:** 2025-08-22

**Authors:** Wanqiu Huang, Hui Wang, Di Wu, Lu Zhang, Jiabing Tong, Minghui Yu, Zegeng Li, Qinjun Yang

**Affiliations:** 1 Anhui University of Chinese Medicine, Hefei, China; 2 The First Affiliated Hospital of Anhui University of Chinese Medicine, Hefei, China; 3 Anhui Province Key Laboratory of the Application and Transformation of Traditional Chinese Medicine in the Prevention and Treatment of Major Pulmonary Diseases, Hefei, China

**Keywords:** Jinshui Liujun decoction, traditional Chinese medicine, chronic obstructive pulmonary disease, trial sequential analysis, meta-analysis

An incomplete Funding statement was provided. The grant number for ‘Key Project of the Scientific Research Program for Higher Education Institutions in Anhui Province’ was not included. Also, the following funder and grant number ‘The Study on the Intervention of Qi Bai Pingfei Capsule on the Pyroptosis of COPD Pulmonary Arterial Adventitial Fibroblasts by Modulating the NLRP3/Casepase-1/GSDMD Pathway (82205074)’ was erroneously omitted.

The correct funding statement reads:

**Funding**

The author(s) declare that financial support was received for the research and/or publication of this article. The National Natural Science Foundation of China (82374399); Key Project of the Scientific Research Program for Higher Education Institutions in Anhui Province (2024AH051047); The Study on the Intervention of Qi Bai Pingfei Capsule on the Pyroptosis of COPD Pulmonary Arterial Adventitial Fibroblasts by Modulating the NLRP3/Casepase-1/GSDMD Pathway (82205074).

In the abstract and main text, the abbreviation for “Jin Shui Liu Jun Decoction” was erroneously provided as ‘JSD’. All occurances of ‘JSD’ have been corrected to “JLD.”

The original article has been updated.

