# Supplementary material for: Efficacy of Jinshui Liujun decoction on chronic obstructive pulmonary disease patients: a systematic review and meta-analysis
Source: Front Pharmacol. 2025 May 21;16:1567452. doi: 10.3389/fphar.2025.1567452 (PMC12133822; doi:10.3389/fphar.2025.1567452)
Supplement: Supplementary file 1 [file Table1.docx]

**Efficacy of Jinshui Liujun Decoction on chronic obstructive pulmonary disease patients: A systematic review and meta-analysis**

**Supplement: content**

| **TABLE S1a. Search strategy in Pubmed** ([https://pubmed. ncbi. nlm. nih. gov/](https://pubmed.ncbi.nlm.nih.gov/)) | |
| --- | --- |
| **COPD** | 1. Pulmonary Disease, Chronic Obstructive[MeSH Terms]  2. Chronic Obstructive Pulmonary Diseases[MeSH Terms]  3. Chronic Obstructive Lung Disease[MeSH Terms]  4. COAD[Title/Abstract]  5. COPD[Title/Abstract]  6. Chronic Obstructive Airway Disease[MeSH Terms]  7. Chronic Obstructive Pulmonary Disease[MeSH Terms]  8. Airflow Obstruction, Chronic[MeSH Terms]  9. Airflow Obstructions, Chronic[MeSH Terms]  10. Chronic Airflow Obstructions[MeSH Terms]  11. Chronic Airflow Obstruction[MeSH Terms]  12. 1 OR 2 OR 3 OR 4 OR 5 OR 6 OR 7 OR 8 OR 9 OR 10 OR 11 |
| **Jinshui Liujun Decoction** | 13. Jin Shui Liu Jun Jian[Title/Abstract]  14. Jinshui Liujun Decoction[Title/Abstract]  15. 13 OR 14 |
| **Study Type** | 16. "Clinical Trial" [Publication Type]  17. "Randomized Controlled Trial" [Publication Type]  18. 16 OR 17 |
| **Combine** | 19. 12 AND 15 AND 17 |

| **TABLE S1b. Search strategy in EMBASE (**<https://www.embase.com/>**)** | |
| --- | --- |
| **COPD** | #1 'chronic obstructive lung disease'/exp  #2 'chronic obstructive pulmonary diseases':ti,ab,kw  #3 'chronic obstructive lung disease':ti,ab,kw  #4 'coad':ti,ab,kw  #5 'pulmonary disease, chronic obstructive'  #6 'copd':ti,ab,kw  #7 'chronic obstructive airway disease':ti,ab,kw  #8 'chronic obstructive pulmonary disease':ti,ab,kw  #9 'airflow obstruction, chronic'  #10 'airflow obstructions, chronic'  #11 'chronic airflow obstructions':ti,ab,kw  #12 'chronic airflow obstruction':ti,ab,kw  #13 #1 OR #2 OR #3 OR #4 OR #5 OR #6 OR #7 OR #8 OR #9 OR #10 OR #11 OR #12 |
| **Jinshui Liujun Decoction** | #14 'jin shui liu jun jian':ab,ti  #15 'jinshui liujun decoction':ab,ti  #16 14 OR 15 |
| **Study Type** | #17 'controlled clinical trial'/exp  #18 'randomized controlled trial'/exp  #19 #17 OR #18 |
| **Combine** | #20 #13 AND #16 AND #19 |

| **TABLE S1c. Search strategy in Web Of Science(**<https://www.webofscience.com/>**)** | |
| --- | --- |
| **COPD** | 1. TS=(copd)  2. TS=(Chronic Obstructive Pulmonary Diseases)  3. TS=(Chronic Obstructive Lung Disease)  4. TI=(COAD)  5. TI=(COPD)  6. TS=(Chronic Obstructive Airway Disease)  7. TS=(Chronic Obstructive Pulmonary Disease)  8. TS=(Chronic Airflow Obstruction)  9. 1 OR 2 OR 3 OR 4 OR 5 OR 6 OR 7 OR 8 |
| **Jinshui Liujun Decoction** | 10. TS=(Jin Shui Liu Jun Jian)  11. TI=(Jin Shui Liu Jun Jian)  12. AB=(Jin Shui Liu Jun Jian)  13. TS=(Jinshui Liujun Decoction)  14. TI=(Jinshui Liujun Decoction)  15. AB=(Jinshui Liujun Decoction)  16. 10 OR 11 OR 12 OR 13 OR 14 OR 15 |
| **Study Type** | 14. TS=(Clinical Trial)  15. TI=(Clinical Trial)  16. AB=(Clinical Trial)  17. TS=(Randomized Controlled Trial)  18. TI=(Randomized Controlled Trial)  19. AB=(Randomized Controlled Trial)  20. 14 OR 15 OR 16 OR 17 OR 18 OR 19 |
| **Combine** | 21. 9 AND 16 AND 20 |

| **TABLE S1d. Search strategy in CENTRAL(**<https://www.proquest.com/>**)** | |
| --- | --- |
| **COPD** | S1. noft(chronic obstructive pulmonary diseases)  S2. mainsubject(pulmonary disease, chronic obstructive)  S3. mainsubject(chronic obstructive lung disease)  S4. ti(COAD)  S5. ti(COPD)  S6. noft(chronic obstructive airways disease)  S7. mainsubject(airflow obstruction, chronic)  S8. mainsubject(airflow obstruction, chronic)  S9. mainsubject(chronic airflow obstruction*)  S10. S1 OR S2 OR S3 OR S4 OR S5 OR S6 OR S7 OR S8 OR S9 |
| **Jinshui Liujun Decoction** | S11. noft(Jin Shui Liu Jun Jian)  S12. noft(Jinshui Liujun Decoction)  S13. S11 OR S12 |
| **Study Type** | S19. mainsubject.Exact("clinical trial")  S20. "clinical trials, phase i as topic"  S21. "clinical trials as topic; humans"  S22. "clinical trials results"  S23. "clinical trials as topic"  S24. "clinical trial results"  S25. "clinical trial design"  S26. "randomized controlled trials as topic; research design"  S27. "clinical trials, phase ii"  S28. "randomized controlled trials"  S29 "clinical trials, phase i"  S30 "clinical trials, phase iv as topic"  S31 "clinical trials protocol"  S32 "clinical trials, phase iv"  S33 "clinical trials, phase ii as topic"  S34 "clinical trials"  S35 "randomized controlled trials as topic"  S36 "clinical trials as topic; female; humans; male; prognosis; survival analysis"  S37 "clinical trials data monitoring committees"  S38 "clinical trials, phase iii as topic"  S39 "clinical trials, phase iii")  S40 noft(Clinical Trial)  S41 mainsubject.Exact("clinical trial"  S42 "clinical trials"  S43 "randomized controlled trials as topic"  S44 "randomized controlled trials as topic; research design"  S45 "randomized controlled trials")  S46 noft(Randomized Controlled Trial)  S47. S19 OR S20 OR S21 OR S22 OR S23 OR S24 OR S25 OR S26 OR S27 OR S28 OR S29 OR S30 OR S31 OR S32 OR S33 OR S34 OR S35 OR S36 OR S37 OR S38 OR S39 OR S40 OR S41 OR S42 OR S43 OR S44 OR S45 OR S46 |
| **Combine** | S48 S10 AND S13 AND S47 |

| **TABLE S1e. Search strategy in SCOUPS(**<https://www.scopus.com/>**)** | |
| --- | --- |
| **COPD** | 1. TITLE-ABS-KEY ( chronic AND obstructive AND pulmonary AND disease* )  2. TITLE-ABS-KEY ( chronic AND obstructive AND lung AND disease* )  3. TITLE-ABS-KEY ( coad )  4. TITLE-ABS-KEY ( copd )  5. TITLE-ABS-KEY ( chronic AND obstructive AND airway AND disease* )  6. TITLE-ABS-KEY ( chronic AND airflow AND obstruction )  7. 1 OR 2 OR 3 OR 4 OR 5 OR 6 |
| **Jinshui Liujun Decoction** | 8. TITLE-ABS-KEY (jin AND shui AND liu AND jun AND jian )  9. TITLE-ABS-KEY (jinshui AND liujun AND decoction )  10. 8 OR 9 |
| **Study Type** | 11. TITLE-ABS-KEY (clinical AND trial)  12. TITLE-ABS-KEY (randomized AND controlled AND trial)  13. 11 OR 12 |
| **Combine** | 14. 7 OR 10 OR 13 |

| **TABLE S1f. Search strategy in Cochrane(**<https://www.cochrane.org/>**)** | |
| --- | --- |
| **COPD** | #1 MeSH descriptor: [Pulmonary Disease, Chronic Obstructive] explode all trees |
| **Jinshui Liujun Decoction** | #2 (Jin Shui Liu Jun Jian):ti,ab,kw  #3 (Jinshui Liujun Decoction):ti,ab,kw  #4 #2 OR #3 |
| **Study Type** | #5 ("clinical trial"):ti,ab,kw  #6 ("randomized controlled trial"):ti,ab,kw  #7 (RCT):ti,ab,kw  #8 #5 OR #6 OR #7 |
| **Combine** | #9 #1 AND #4 AND #8 |

| **TABLE S1g. ProQuest(**<https://www.proquest.com>**)** | |
| --- | --- |
| **COPD** | S1 noft(chronic obstructive pulmonary diseases) OR mainsubject(pulmonary disease, chronic obstructive) OR mainsubject(chronic obstructive lung disease) OR ti(COAD) OR ti(COPD) OR noft(chronic obstructive airways disease) OR mainsubject(airflow obstruction, chronic) OR mainsubject(airflow obstruction, chronic) OR mainsubject(chronic airflow obstruction*) |
| **Jinshui Liujun Decoction** | S2 noft(Jin Shui Liu Jun Jian) OR noft(Jinshui Liujun Decoction) |
| **Study Type** | S3 mainsubject.Exact("clinical trial" OR "clinical trials, phase i as topic" OR "clinical trials as topic; humans" OR "clinical trials results" OR "clinical trials as topic" OR "clinical trial results" OR "clinical trial design" OR "randomized controlled trials as topic; research design" OR "clinical trials, phase ii" OR "randomized controlled trials" OR "clinical trials, phase i" OR "clinical trials, phase iv as topic" OR "clinical trials protocol" OR "clinical trials, phase iv" OR "clinical trials, phase ii as topic" OR "clinical trials" OR "randomized controlled trials as topic" OR "clinical trials as topic; female; humans; male; prognosis; survival analysis" OR "clinical trials data monitoring committees" OR "clinical trials, phase iii as topic" OR "clinical trials, phase iii") OR noft(Clinical Trial) OR mainsubject.Exact("clinical trial" OR "clinical trials" OR "randomized controlled trials as topic" OR "randomized controlled trials as topic; research design" OR "randomized controlled trials") OR noft(Randomized Controlled Trial) |
| **Combine** | S4 S1 AND S2 AND S3 |

| **TABLE S1g. Search strategy in Commonly Used Databases for Research in China** | |
| --- | --- |
| **CNKI (**<https://www.cnki.net/>**)** | (SU=Jin Shui Liu Jun Jian + Jia Wei Jin Shui Liu Jun Jian + Liu Jun Jian) AND (SU=Chronic Obstructive Pulmonary Disease + Chronic Obstructive Pulmonary Illness + Chronic Obstructive Pulmonary Disorders + COPD) AND (AB=Randomized + Controlled + Clinical) |
| **WanFangdata(**<https://www.wanfangdata.com.cn/>**)** | Subject: ("Jin Shui Liu Jun Jian" OR "Jia Wei Jin Shui Liu Jun Jian" OR "Liu Jun Jian") AND Subject: ("Chronic Obstructive Pulmonary Disease" OR "Chronic Obstructive Pulmonary Illness" OR "Chronic Obstructive Pulmonary Disorders" OR "COPD") AND Abstract: ("Randomized" OR "Controlled" OR "Clinical") |
| **VIP**[(http://www.cqvip.com/]((http:/www.cqvip.com/)) | ((((R=Jin Shui Liu Jun Jian OR R=Jia Wei Jin Shui Liu Jun Jian) OR R= Liu Jun Jian) AND (((R= Chronic Obstructive Pulmonary Disease OR R= Chronic Obstructive Pulmonary Illness) OR R= Chronic Obstructive Pulmonary Disorders) OR R=COPD)) AND ((R= Randomized OR R= Controlled) OR R= Clinical)) |
| **Sinomed**(<http://www.sinomed.ac.cn>) | ("Randomized"[Abstract:Intelligent]OR"Controlled"[Abstract:Intelligent]OR"Clinical"[Abstract:Intelligent])AND("ChronicObstructivePulmonaryDisease"[Unweighted:Expanded])AND("JinShuiLiuJunJian"[Unweighted:Expanded]) |
